# Tumor-Associated Neutrophils Can Predict Lymph Node Metastasis in Early Gastric Cancer

**DOI:** 10.3389/fonc.2020.570113

**Published:** 2020-09-21

**Authors:** Yaohui Wang, Jing Zhai, Tiancheng Zhang, Shutang Han, Yifen Zhang, Xuequan Yao, Lizong Shen

**Affiliations:** ^1^Department of Pathology, Jiangsu Province Hospital of Chinese Medicine, Affiliated Hospital of Nanjing University of Chinese Medicine, Nanjing, China; ^2^Department of Surgical Oncology, Jiangsu Province Hospital of Chinese Medicine, Affiliated Hospital of Nanjing University of Chinese Medicine, Nanjing, China; ^3^Digestive Endoscopy Center, Jiangsu Province Hospital of Chinese Medicine, Affiliated Hospital of Nanjing University of Chinese Medicine, Nanjing, China

**Keywords:** early gastric cancer, tumor-associated neutrophils, lymph node metastasis, cancer-associated fibroblasts, IL-8

## Abstract

The consensus of endoscopic therapy for early gastric cancer (EGC) mainly depends on its clinicopathological features. However, the roles of tumor-associated neutrophils (TANs) in EGC remain uncertain. Here, we explored its predictive role for lymph node metastasis (LNM) in EGC. Three hundred twenty-two patients who underwent radical gastrectomy for EGC were enrolled. Preoperative peripheral blood was used to analyze the neutrophil-to-lymphocyte ratio (NLR), and the different status of TANs was determined by hematoxylin-and-eosin staining (H&E) and immunohistochemistry (IHC). TANs, rather than NLR, were positively associated with tumor size, Lauren classification, lymphovascular invasion (LVI), and LNM. Univariate analysis revealed that TANs were associated with LNM as well as tumor size, depth of invasion, Lauren classification, histological classification, LVI, and perineural invasion. In addition to histological classification and LVI, TANs were found to be an independent risk factor for LNM in EGC (*P* = 0.013). Stratification analysis by depth of invasion showed LVI in SM1 tumor, and both LVI and TANs (*P* = 0.042) in SM2 tumor were independent risk factors for LNM. In conclusion, TANs in EGC can predict LNM, and TANs may help to estimate LNM precisely in addition to the current criteria.

## Introduction

Gastric cancer is one of the most lethal malignant diseases worldwide, especially in China, ranking second in the incidence and third in the mortality of all malignant tumors (1, 2). The incidence of early gastric cancer (EGC) has been increasing in the past decades with the enhanced public awareness of this disease and the development of screening technologies. Endoscopic therapy, including endoscopic mucosal resection (EMR) and endoscopic submucosal dissection (ESD), has been generally recognized to be appropriate for EGC, and the prognosis of EGC has been improved (3). However, concerns about the concurrent lymph node metastasis (LNM) have been plaguing endoscopic therapy for EGC (4). There are several established consensuses for this issue; clinicopathological features, such as tumor size, invasion depth, histological classification, ulceration, and lymphovascular infiltration (LVI), have been used to assess the risk of LNM in EGC (5, 6). Currently, three evaluation categories (absolute, expanded, and relative indications or endoscopic curability A, B, and C) are used to guide endoscopic therapy for EGC depending on the incidence of LNM and completeness of the primary tumor removal (5, 6). Actually, endoscopic therapy has some pitfalls for EGC according to these current guidelines. Additional surgical resection revealed that LNM was detected in only 5% of patients who did not meet the criteria of curative endoscopic resection (7, 8). However, it has been demonstrated that ~0.2% patients with absolute indication and 0.7% patients with expanded indication encountered simultaneous LNM (4). Therefore, seeking a more precise indicator for LNM is compelling for EGC.

The abovementioned consensus mainly depends on morphological characteristics and histological classification of tumors. It has been established that gastric cancer is a highly heterogeneous tumor, and tumor microenvironment (TME) plays an important role in stomach carcinogenesis, tumor progression, therapeutic response, and the prognosis (9). Complex and diverse cell components are found in TME as well as tumor cells, including fibroblasts, immune cells, pericytes, and endothelial cells (10). Neutrophils infiltrating in the TME, known as tumor-associated neutrophils (TANs), have been shown to be involved in tumorigenesis, angiogenesis, and tumor metastasis (11, 12). Cancer-associated fibroblasts (CAFs) are the predominant cell type in the tumor-associated stroma and contribute to tumorigenesis by secreting growth factors, modifying the extracellular matrix, supporting angiogenesis, suppressing antitumor immune responses, and fostering resistance to therapy (13). We have revealed previously that CAFs in gastric cancer tissues produce massive IL-8, which results in chemoresistance, and CAFs are associated with poor prognosis for advanced gastric cancer patients (14). IL-8 is the prominent chemokine of neutrophils, and it may recruit neutrophils to tumor tissues *via* interaction with CXCR1/2 in neutrophils (15). However, whether TANs and/or CAFs are associated with LNM in EGC remains unknown.

In this study, we performed a retrospective study with 322 cases of EGC, who underwent radical gastrectomy with curative intent, to investigate the predictive roles of TANs and/or CAFs for LNM. We showed that TANs and CAFs were associated with clinicopathological features in EGC, including tumor size, depth of invasion, Lauren classification, lymphovascular invasion (LVI), and LNM. Univariate analysis revealed that TANs and CAFs were associated with LNM as well as tumor size, depth of invasion, Lauren classification, histological classification, LVI, and perineural invasion. However, histological classification, LVI, and TANs were the independent risk factors for LNM with multivariate analysis. TAN infiltration was consistent with mature CAFs. Our results identified that TANs in EGC can predict LNM and that TANs may help to estimate LNM precisely in addition to the current criteria.

## Materials and Methods

### Patients

As shown in a CONSORT diagram, a consecutive series of 322 patients with EGC from January 2011 to December 2017 in the Affiliated Hospital of Nanjing University of Chinese Medicine were enrolled in the study. All these patients underwent open or laparoscopic D2 radical gastrectomy with curative intent and were pathologically diagnosed with early gastric adenocarcinoma according to the American Joint Committee on Cancer (AJCC) criteria. EGC was defined as a tumor invading the mucosa or submucosa (pT1). All these patients had not received preoperative chemotherapy or radiotherapy, and all these patients received follow-up for overall survival (OS) by telephone or subsequent consultation with a cutoff date of December 2019. The follow up time was 24–108 months (median: 53 months).

The stomach was anatomically divided into three portions: the upper third, the middle third, and the lower third. The depth of tumor infiltration was divided into three groups: intramucosa (tumor infiltration confined in the mucosa lamina propria or muscularis mucosae), SM1 (tumor infiltration confined in the superficial submucosal layer, <500 μm from the muscularis mucosae), and SM2 (tumor infiltration confined in the deep submucosal layer, more than 500 μm from the muscularis mucosae). Lauren classification includes intestinal, diffuse, mixed, and undefined type according to the fifth edition of WHO criteria (16). The clinicopathological features were collected, including gender, age (<65 or ≥65); tumor location; tumor size (<2, 2–2.9, or ≥3 cm); macroscopic type (elevated, flat, or depressed); depth of tumor infiltration (intramucosa, SM1, or SM2); histological classification (well, moderately, or poorly differentiated); the presence of LVI, perineural invasion, and *Helicobacter pylori* infection; and the status of LNM. All these pathological features were reviewed for this study by two experienced pathologists. The samples were obtained following written consent according to an established protocol approved by the Institutional Review Board of Nanjing University of Chinese Medicine. This study was also in compliance with the Declaration of Helsinki.

**Figure:**
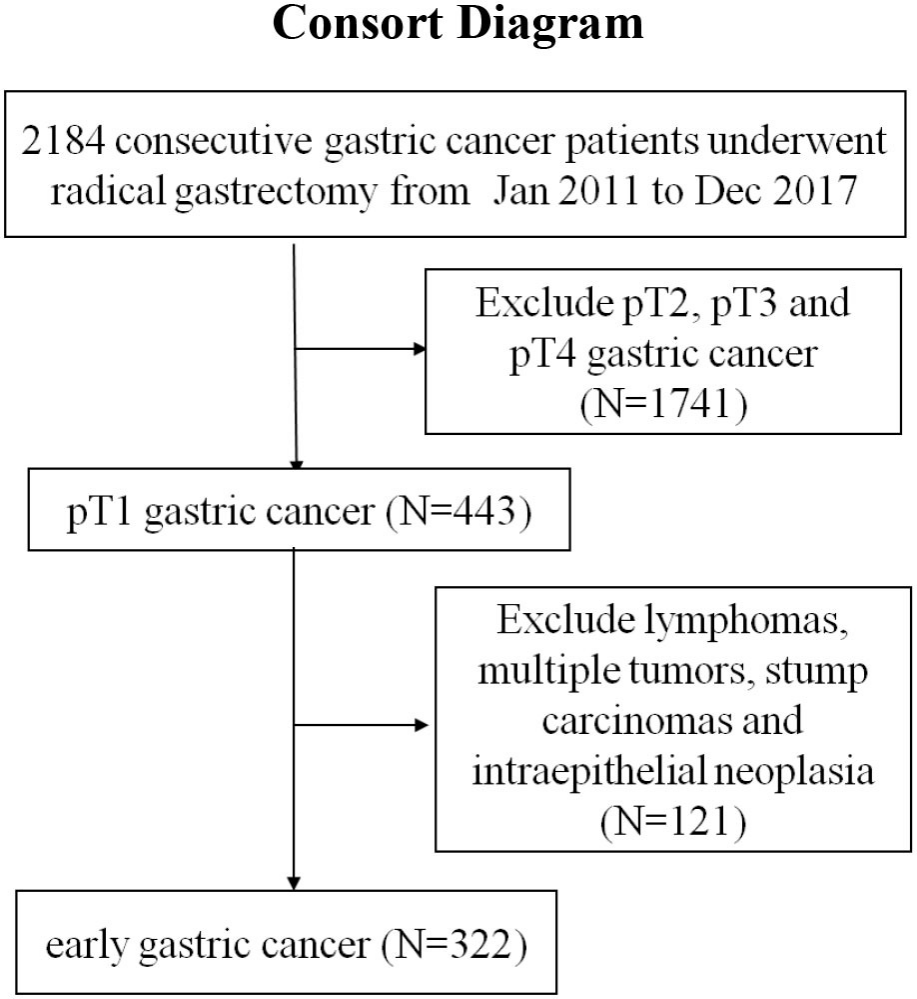


### Blood Neutrophil-to-Lymphocyte Ratio (NLR) Analysis

The neutrophil counts and lymphocyte counts in the pretherapeutic blood routine of these patients were collected. The NLR was defined as the neutrophil count divided by the lymphocyte count. The median value of NLR in these patients was used as the cutoff of NLR, and these patients were divided into the high-NLR group and the low-NLR group.

### TAN Analysis

The TANs were determined pathologically by H&E staining and immunohistochemistry (IHC). A rabbit polyclonal anti-myeloperoxidase (MPO) antibody (Cell Signaling Technology, MA, USA) was used, and H&E staining or IHC was performed on paraffin-embedded formalin-fixed tissues according to standard protocols. Neutrophil was defined as lobulated nuclei, and the cytoplasm was rich in reddish granules. Neutrophils engaged within the tumor tissue (including the neoplastic parenchyma and the tumor stroma) were referred to as TANs (17). All the slides containing tumor tissue were observed at low power, and the section with the most apparent neutrophilic aggregates within the tumor tissue was selected. Ten non-overlapping high-power fields (HPFs, 400-fold magnification, field diameter 0.55 μm) were observed continuously. However, the areas closely adjacent to mucosal erosion, ulcer, or infarct-like necrosis of tumor were excluded for neutrophil counting. Patients were divided into high- and low-TAN infiltration groups, based on a median TAN number of 10 per HPF in primary tumors.

### CAF Analysis

CAFs constitute the predominant stromal component in many types of malignancies. Due to their aforementioned interrelation with neutrophils, we performed CAF analysis pathologically by H&E staining and IHC. A mouse monoclonal anti-α-smooth muscle actin (α-SMA) antibody (Cell Signaling Technology, MA, USA) was used. CAFs were defined as fibroblast-like cells proliferating around the tumor parenchyma (18). Patients were classified as high- or low-CAF phenotype depending on the percentage of CAFs in tumor stroma. According to the report of Lee et al. (19), cases were defined as high-CAF phenotype if CAFs accounted for more than 50% of tumor stroma; otherwise, cases were defined as low-CAF phenotype. However, 12 cases diagnosed with mucinous adenocarcinoma were not included in the CAF study due to the extracellular mucin pools instead of obvious cells. Ueno et al. (20) reported that CAFs can be classified into mature or immature CAFs. Mature CAFs are defined as fine elongated collagen fibers occupying the background predominantly, which contains long and thin spindle fibroblast cells with fewer weak eosinophilic cytoplasm, dense nuclei, and inconspicuous nucleoli. Immature CAFs are defined as mucoid without obvious collagen fibers or a few keloid-like collagens, which contains plump spindle- or stellate-shaped fibroblast cells with weak basophilic cytoplasm, oval and vacuolar nuclei, and prominent nucleoli. The expression of α-SMA in mature CAFs was weaker than that in immature CAFs. We evaluated the maturity of CAFs in the high-CAF group.

### Detection of IL-8 in Tumor Tissues

The expression of IL-8 in gastric tissue specimens was detected by IHC according to standard protocols. A mouse monoclonal anti-IL-8 antibody (Abcam, Cambridge, UK) was used.

### Statistical Analysis

Differences and relationships between groups with continuous or categorical variables were statistically compared with Student *t*, χ^2^, Fisher's exact test, and Spearman correlation analysis using SPSS software (version 22.0, SPSS Inc., Chicago, IL). The OSs were calculated using the Kaplan–Meier method. The log-rank test was used to compare the difference between these groups. A multivariate logistic regression analysis was performed to identify the independent risk factors of LNM. *P* < 0.05 were considered to be statistically significant.

## Results

### TANs and CAFs Were Associated With Clinicopathological Features in EGC

**Figure 1:**
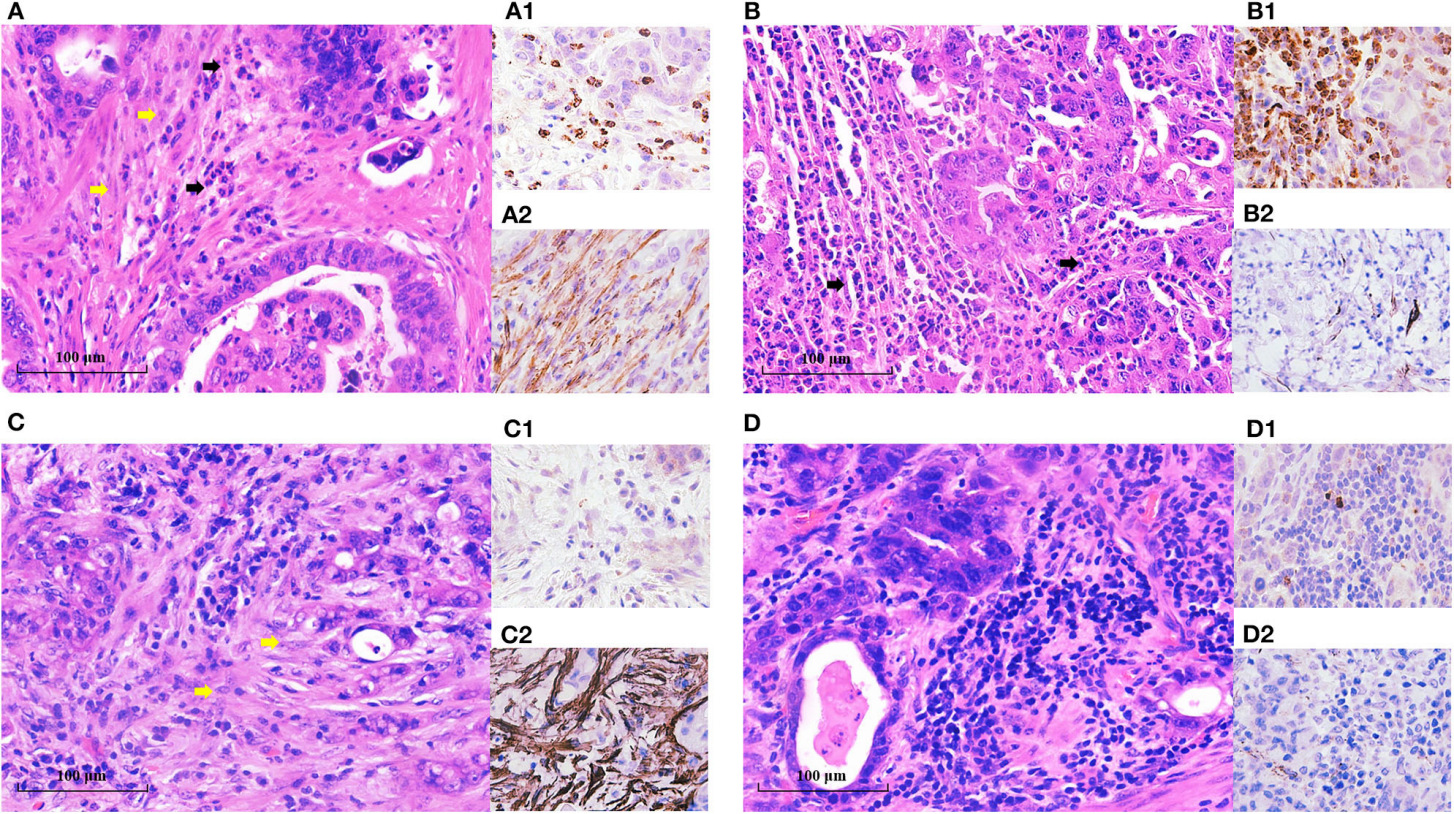
TANs and CAFs in gastric cancer tissues. TANs and CAFs were detected in the tumor stroma of all these EGC patients by H&E staining and IHC. **(A)** Both the high-TAN and high-CAF groups: more than 10 TANs per HPF (black arrow) were detected, and CAFs (yellow arrow) accounted for more than 50% of tumor stroma. **(B)** The high-TAN group with low CAFs: more than 10 TANs per HPF (black arrow) were detected, and CAFs accounted for <50% of tumor stroma. **(C)** The low-TAN group with high CAFs: <10 TANs per HPF were detected, and CAFs (yellow arrow) accounted for more than 50% of tumor stroma. **(D)** The low-TAN group with low CAFs: <10 TANs per HPF were detected, and CAFs accounted for <50% of tumor stroma. (**A1**, **B1**, **C1**, **D1**) TANs using MPO staining. (**A2**, **B2**, **C2**, **D2**) CAFs using α-SMA staining.

The clinicopathological features of these enrolled patients were listed in Table 1. We first investigated the clinicopathological significance of TANs and CAFs. As shown in Figure 1, TANs and CAFs were detected in the tumor stroma of all these EGC patients, and 44% (130/322) constituted the high-TAN group, while 45.2% (140/310) were classified into the high-CAF group (Table 1). TANs were associated with the tumor size (*P* = 0.036), Lauren classification (*P* = 0.001), LVI (*P* = 0.005), and LNM (*P* = 0.000) significantly (Table 1). CAFs were closely correlated with tumor size (*P* = 0.003), depth of invasion (*P* = 0.000), Lauren classification (*P* = 0.043), LVI (*P* = 0.000), perineural invasion (*P* = 0.000), and LNM (*P* = 0.000) (Table 1). However, there was no remarkable association between TAN infiltration and CAFs (*r* = 0.059, *P* = 0.351) (Supplementary Table S1).

**Table 1:** The clinical significance of TANs and CAFs in the EGC tissues.

**Clinicopathological features**		**TANs**		**χ^**2**^**	***P***	**CAFs**		**χ^**2**^**	***P***
		**Low** **(*n* = 192) (%)**	**High** **(*n* = 130) (%)**			**Low** **(*n* = 170) (%)**	**High** **(*n* = 140) (%)**		
Gender	Male	128 (58.4)	91 (41.6)	0.257	0.545	109 (52.2)	100 (47.8)	1.550	0.182
	Female	64 (62.1)	39 (37.9)			61 (60.4)	40 (39.6)		
Age (year)	<65	130 (62.2)	79 (37.8)	1.348	0.234	119 (58.6)	84 (41.4)	2.969	0.072
	≥65	62 (54.9)	51 (45.1)			51 (47.7)	56 (52.3)		
Tumor location in the stomach	Upper third	44 (62.9)	26 (37.1)	5.053	0.079	43 (64.2)	24 (35.8)	3.896	0.148
	Middle third	53 (68.8)	24 (31.2)			41 (56.9)	31 (43.1)		
	Lower third	95 (54.3)	80 (45.7)			86 (50.3)	85 (49.7)		
Tumor size (cm)	<2 cm	111 (65.7)	58 (34.3)	6.645	0.036	105 (64.0)	59 (36.0)	11.867	0.003
	2–2.9	51 (56.7)	39 (43.3)			38 (44.7)	47 (55.3)		
	≥3	30 (47.6)	33 (52.4)			27 (44.3)	34 (55.7)		
Macroscopic type	Elevated	10 (38.5)	16 (61.5)	5.563	0.056	10 (43.5)	13 (56.5)	4.745	0.086
	Flat	31 (66.0)	16 (34.0)			32 (68.1)	15 (31.9)		
	Depressed	151 (60.6)	98 (39.4)			128 (53.3)	112 (46.7)		
Depth of invasion	Intramucosa	96 (61.5)	60 (38.5)	1.894	0.406	142 (92.8)	11 (7.2)	217.004	0.000
	SM1	21 (50.0)	21 (50.0)			17 (41.5)	24 (58.5)		
	SM2	75 (60.5)	49 (39.5)			11 (9.5)	105 (90.5)		
Lauren classification	Intestinal	117 (62.9)	69 (37.1)	15.472	0.001	108 (58.4)	77 (41.6)	8.034	0.043
	Diffuse	37 (75.5)	12 (24.5)			28 (62.2)	17 (37.8)		
	Mixed	33 (45.2)	40 (54.8)			30 (45.5)	36 (54.5)		
	Undefined	5 (35.7)	9 (64.3)			4 (28.6)	10 (71.4)		
Histological classification	Well	32 (74.4)	11 (25.6)	5.597	0.060	23 (56.1)	18 (43.9)	0.054	0.987
	Moderately	87 (54.7)	72 (45.3)			84 (54.9)	69 (45.1)		
	Poorly	73 (60.8)	47 (39.2)			63 (54.3)	53 (45.7)		
Lymphovascular invasion	Absence	170 (63.2)	99 (36.8)	7.773	0.005	166 (63.6)	95 (36.4)	48.983	0.000
	Presence	22 (41.5)	31 (58.5)			4 (8.2)	45 (91.8)		
Perineural invasion	Absence	185 (60.1)	123 (39.9)	0.223	0.579	170 (57.2)	127 (42.8)	14.247	0.000
	Presence	7 (50.0)	7 (50.0)			0 (0.0)	13 (100.0)		
*Helicobacter pylori* infection	Absence	141 (62.9)	83 (37.1)	2.930	0.084	119 (54.8)	98 (45.2)	0.000	1.000
	Presence	51 (52.0)	47 (48.0)			51 (54.8)	42 (45.2)		
Lymph node metastasis	Absence	175 (64.1)	98 (35.9)	13.729	0.000	164 (61.9)	101 (38.1)	34.683	0.000
	Presence	17(34.7)	32(65.3)			6 (13.3)	39 (86.7)		
Neutrophil count	average ± SD	3.35 ± 1.36	3.45 ± 1.39	*F* = 0.017	0.505	3.31 ± 1.27	3.48 ± 1.46	*F* = 3.048	0.254
NLR	Low ( ≤ 1.9)	102 (63.4)	59 (36.6)	1.561	0.211	92 (59.7)	62 (40.3)	2.588	0.088
	High (>1.9)	90 (55.9)	71 (44.1)			78 (50.0)	78 (50.0)		

*TANs, tumor-associated neutrophils; CAFs, cancer-associated fibroblasts; NLR, neutrophil-to-lymphocyte ratio*.

We also evaluated the clinical relevance of the preoperative neutrophil counts and NLR of these patients. The neutrophil counts ranged from 0.80 × 10^9^ to 10.12 × 10^9^/L with an average of 3.39 ± 1.37 × 10^9^/L, while the NLR values varied from 0.62 to 11.8 with a median value of 1.9. Therefore, 1.9 was used as the cutoff value for low NLR or high NLR. As shown in Supplementary Table S2, both the neutrophil counts and the NLR showed no significant association with the clinicopathological features except patient gender, which was not consistent with locally advanced gastric cancer (21).

The OSs between different TAN or CAF groups were also investigated respectively. A total of 43 patients died in this patient cohort (11–55 months, median: 33 months). The low-TAN group or the low-CAF group showed slight improvement in OS compared with the high-TAN group or the high-CAF group; however, it did not reach significant significance (*P* = 0.069 for low- and high-TAN groups, *P* = 0.649 for low- and high-CAF groups) (Supplementary Figure S1).

### Risk Factors for LNM by Univariate and Multivariate Analysis

The presence of LNM remains a major concern in endoscopic therapy for EGC; therefore, we investigated the risk factors associated with LNM in our patient cohort undergoing radical surgery. The average number of removed regional lymph nodes was 21.4 per case (ranging from 12 to 68). The incidence of LNM was 15.2% (49/322) in this whole cohort, and it was 5.8% (9/156), 14.3% (6/42), and 27.4% (34/124) in the intramucosal tumor, SM1 tumor, and SM2 tumor, respectively.

**Figure 2:**
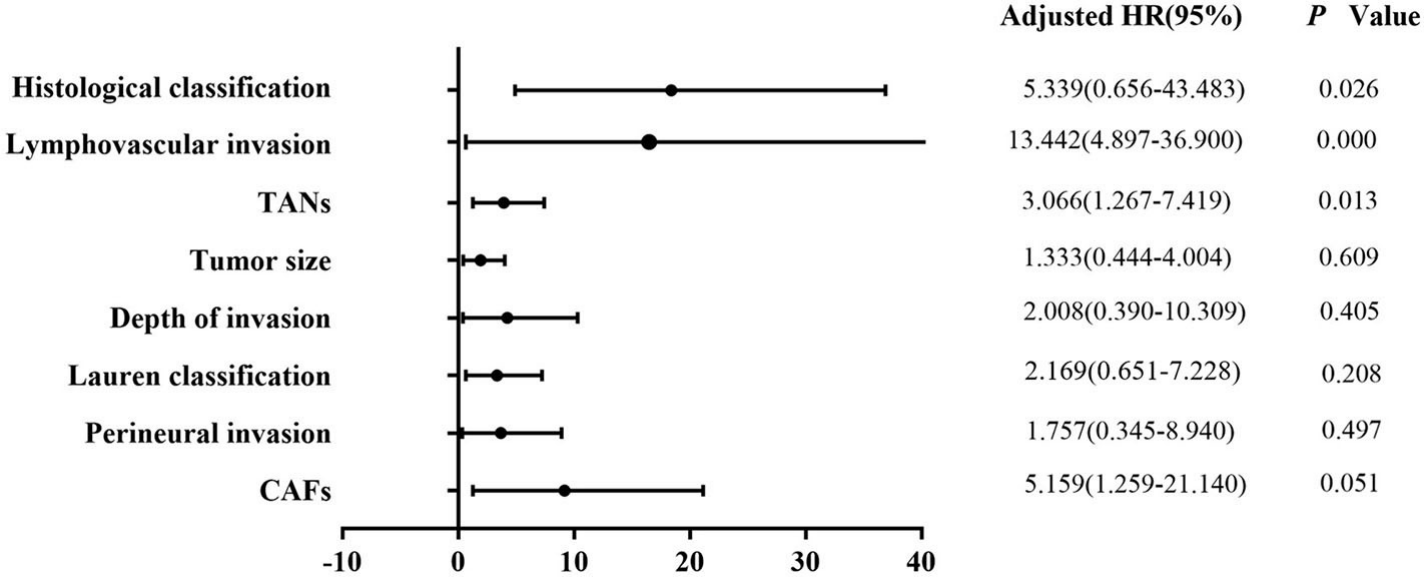
Multivariate logistic regression analysis of potential risk factors for LNM in patients with EGC. Histological classification (*P* = 0.026), LVI (*P* = 0.000), and TANs (*P* = 0.013) were the independent risk factors for LNM in the patients with EGC.

**Figure 3:**
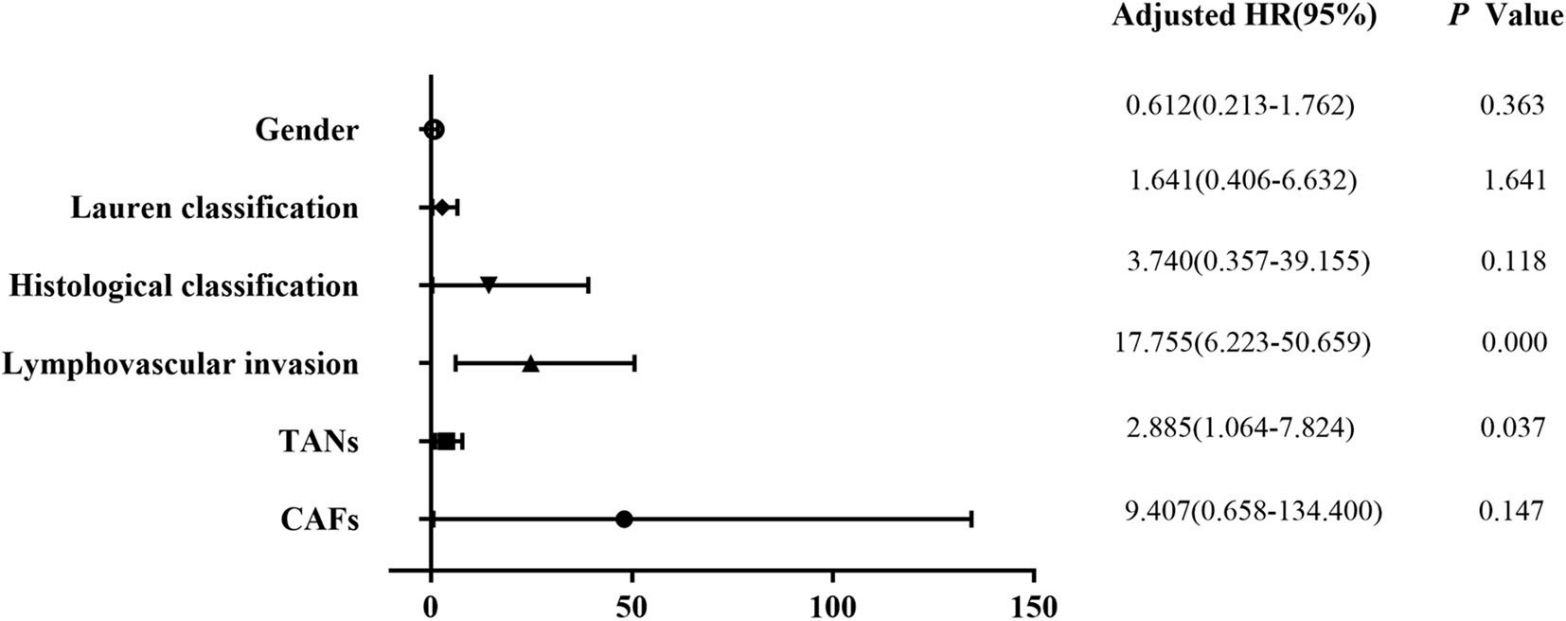
Multivariate logistic regression analysis of potential risk factors for LNM in patients with submucosal EGC. Both LVI (*P* = 0.000) and TANs (*P* = 0.037) were the independent factors for LNM in patients with submucosal EGC.

**Figure 4:**
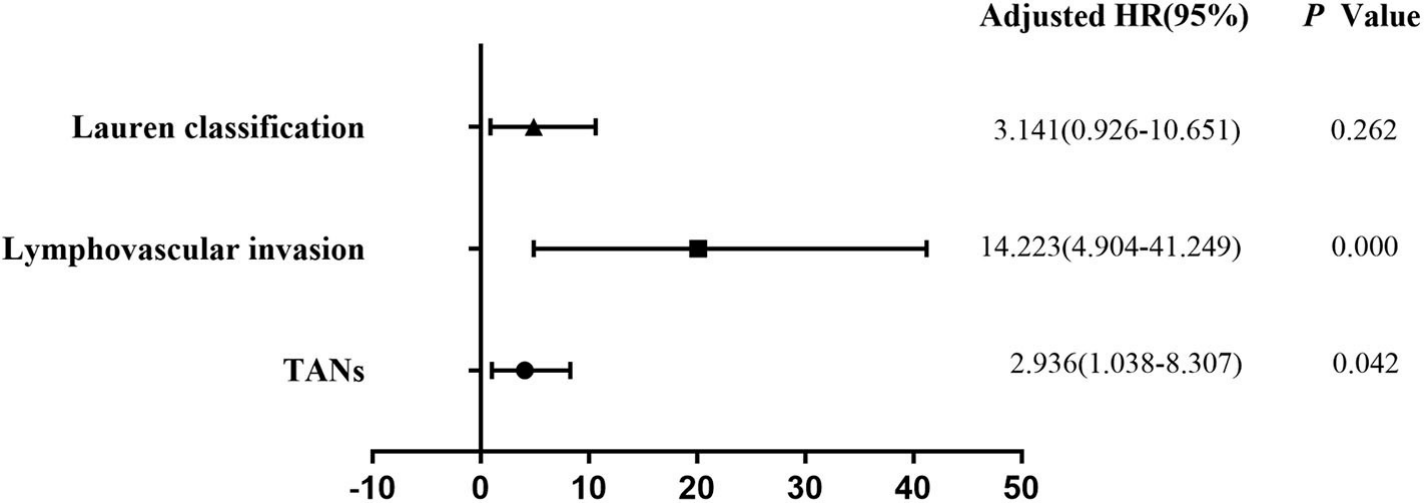
Multivariate logistic regression analysis of potential risk factors for LNM in patients with SM2 EGC. Stratification analysis indicated that both LVI (*P* = 0.000) and TANs (*P* = 0.042) were the independent factors for SM2 tumors.

Univariate analysis revealed that tumor size (*P* = 0.003), depth of invasion (*P* = 0.000), Lauren classification (*P* = 0.000), histological classification (*P* = 0.001), LVI (*P* = 0.000), perineural invasion (*P* = 0.011), TANs (*P* = 0.000), and CAFs (*P* = 0.000) were associated with LNM significantly in the 322 patients (Table 2). However, multivariate analysis indicated that histological classification (*P* = 0.026), LVI (*P* = 0.000), and TANs (*P* = 0.013) were the independent risk factors for LNM (Figure 2). We performed stratification analysis by depth of invasion to identify the specific factors for LNM in different stages of EGC. Intramucosal tumors are the best indication for endoscopic therapy (5, 6). However, tumor size (*P* = 0.038), Lauren classification (*P* = 0.004), histological classification (*P* = 0.015), TANs (*P* = 0.028), and CAFs (*P* = 0.002) were associated with LNM of intramucosal tumors (Supplementary Table S3), and only CAF was the independent risk factor (*P* = 0.049) (Supplementary Table S4). For submucosal tumors, the risk factors included Lauren classification (*P* = 0.001), histological classification (*P* = 0.004), LVI (*P* = 0.000), TANs (*P* = 0.003), and CAFs (*P* = 0.003) (Supplementary Table S5); however, both LVI (*P* = 0.000) and TANs (*P* = 0.037) were the independent factors (Figure 3). Further stratification analysis indicated that both LVI (*P* = 0.000) and TANs (*P* = 0.042) were the independent factors for SM2 tumors (Figure 4), and only LVI was the independent factor for SM1 tumors (*P* = 0.041) (Supplementary Table S6). Collectively, our results indicated that TANs, in addition to the established factor LVI (5, 6), can predict LNM in EGC, especially in SM2 tumor.

**Table 2:** Clinicopathological features associated with LNM in EGC patients.

**Clinicopathological features**		**LNM**		**χ^**2**^**	***P***
		**Present** **(*n* = 49) (%)**	**Absent** **(*n* = 273) (%)**		
Gender	Male	30 (13.7)	189 (86.3)	1.220	0.318
	Female	19 (18.4)	84 (81.5)		
Tumor location in the stomach	Upper third	7 (10.0)	63 (90.0)	1.988	0.370
	Middle third	12 (15.6)	65 (84.4)		
	Lower third	30 (17.1)	145 (82.9)		
Age (year)	<65	26 (12.4)	183 (87.6)	3.549	0.073
	≥65	23 (20.3)	90 (79.6)		
Tumor size (cm)	<2	15 (8.9)	154 (91.1)	11.923	0.003
	2–2.9	18 (20.0)	72 (80.0)		
	≥3	16 (25.4)	47 (74.6)		
Macroscopic type	Elevated	5 (19.2)	21 (80.8)	2.102	0.350
	Flat	4 (8.5)	43 (91.5)		
	Depressed	40 (16.1)	209 (83.9)		
Depth of invasion	Intramucosal	9 (5.8)	147 (94.2)	25.132	0.000
	SM1	6 (14.3)	36 (85.7)		
	SM2	34 (27.4)	90 (72.6)		
Lauren classification	Intestinal	14 (7.5)	172 (92.5)	29.467	0.000
	Diffuse	7 (14.3)	42 (85.7)		
	Mixed	25 (34.2)	48 (65.8)		
	Not defined	3 (21.4)	11 (78.6)		
Histological classification	Well	2 (4.7)	41 (95.3)	15.146	0.001
	Moderately	17 (10.7)	142 (89.3)		
	Poorly	30 (25.0)	90 (75.0)		
Lymphovascular invasion	Absence	18 (6.7)	251 (93.3)	91.795	0.000
	Presence	31 (58.5)	22 (41.5)		
Perineural invasion	Absence	43 (14.0)	265 (86.0)	8.640	0.011
	Presence	6 (42.9)	8 (57.1)		
*Helicobacter pylori* infection	Absence	33 (14.7)	192 (85.3)	0.134	0.737
	Presence	16 (16.5)	81 (83.5)		
TANs	Low	17 (8.9)	175 (91.1)	14.669	0.000
	High	32 (24.6)	98 (75.4)		
CAFs	High	39 (27.9)	101 (72.1)	36.499	0.000
	Low	6 (3.5)	164 (96.4)		
Neutrophil count	average ± SD	3.23 ± 1.43	3.42 ± 1.36	*F* = 0.129	0.393
NLR	Low ( ≤ 1.9)	25 (15.5)	136 (84.5)	0.000	1.000
	High (>1.9)	24 (14.9)	137 (85.1)		

*LNM, lymph node metastasis; TANs, tumor-associated neutrophils; CAFs, cancer-associated fibroblasts; NLR, neutrophil-to-lymphocyte ratio*.

### Correlation Between TANs and CAFs in EGC Tumor Tissues

**Figure 5:**
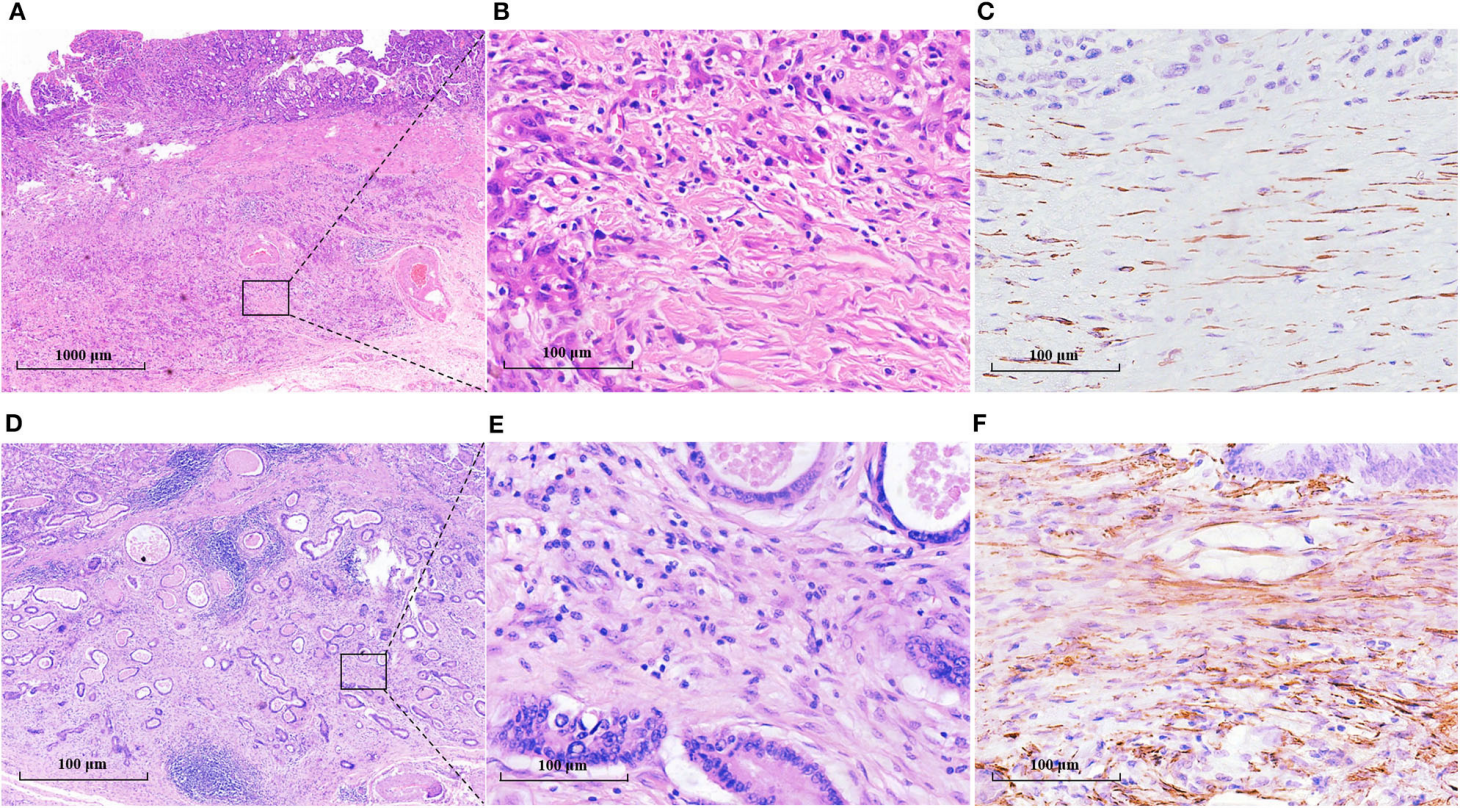
The morphological characteristics and α-SMA expression of mature and immature CAFs in tumor tissues. **(A–C)** Mature CAFs: the cancer invaded the deep submucosa (SM2), and mature CAFs mainly constituted the stroma **(A)**. **(B)** was the amplification of the black frame in **A** and showed thin spindle fibroblasts with fine elongated collagen fibers. **C** showed the mature CAFs with weak positive α-SMA staining. **(D–F)** Immature CAFs: the cancer invaded the deep submucosa (SM2), and immature CAFs mainly constituted the stroma **(D)**. **(E)** was the amplification of the black frame in **(D)** and showed plump spindle fibroblast cells without obvious collagen fibers. **(F)** showed the immature CAFs with a highly positive expression of α-SMA.

To probe the potential mechanisms underlying neutrophil infiltration in gastric cancer tissues, we evaluated the correlation between TANs and CAFs, because CAFs can produce IL-8 and IL-8 is one of the prominent chemokines for neutrophils. The prevalence of the high-CAF subgroup was 7.2% for intramucosal tumors and increased to 58.5% for SM1 tumors and 90.5% for SM2 tumors (Table 1). However, the prevalence of CAFs had nothing to do with that of TANs in the whole patient cohort or in the patients with SM tumors (Supplementary Table S1). We further studied the maturity of CAFs and their relationship with TANs. Figure 5 shows the morphological characteristics of mature and immature CAFs in tumor tissues. The mature CAFs were associated with TANs in all SM tumors (*r* = 0.180, *P* = 0.046) (Table 3) and in SM2 tumors (*r* = 0.215, *P* = 0.034) (Table 4), but they had no correlation in SM1 tumors (Supplementary Table S7). Importantly, the mature CAFs were significantly related to LNM in SM2 tumors (*P* = 0.004) (Supplementary Table S8).

**Table 3:** Correlation between TANs and mature or immature CAFs in submucosal gastric cancer tissues (*n* = 129).

**SM**		**CAFs**		**χ^**2**^**	***r***	***P***
		**Mature**	**Immature**			
TANs	High	15	39	3.319	0.180	0.046
	Low	10	65			

*TANs, tumor-associated neutrophils; CAFs, cancer-associated fibroblasts*.

**Table 4:** Correlation between TANs and mature or immature CAFs in SM2 gastric cancer tissues (*n* = 105).

**SM2**		**CAFs**		**χ^**2**^**	***r***	***P***
		**Mature**	**Immature**			
TANs	High	14	27	3.868	0.215	0.034
	Low	10	54			

*TANs, tumor-associated neutrophils; CAFs, cancer-associated fibroblasts*.

**Figure 6:**
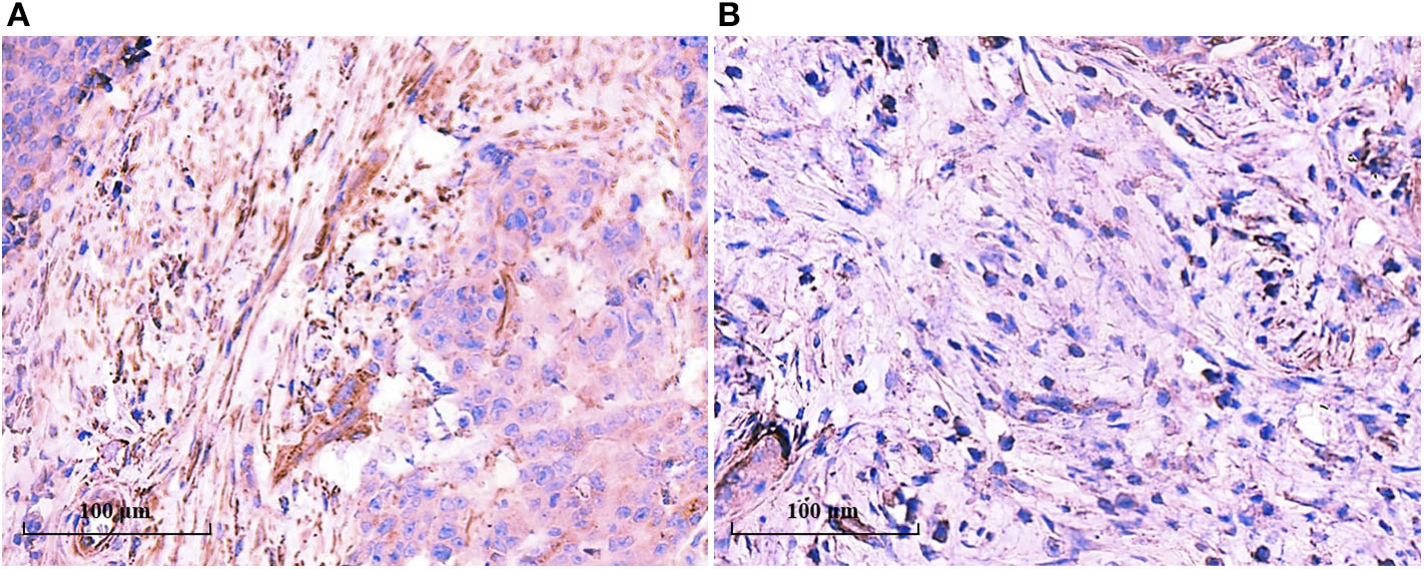
IL-8 was highly expressed in mature CAFs. IL-8 expression in the tumor tissues was determined by IHC, and IL-8 was detected in tumor stroma rather than in tumor cells. IL-8 was highly expressed in mature CAFs **(A)**, while it was seldom detected in immature CAFs **(B)**.

The above results hinted that TAN infiltration was consistent with mature CAFs. We examined the IL-8 expression in the tumor tissues by IHC. As shown in Figure 6, IL-8 was highly expressed in mature CAFs rather than in immature CAFs, and IL-8 expression was consistent with TAN distribution, which supported the notion that CAF-derived IL-8 recruits neutrophils in tumor tissues and promotes tumor progression subsequently.

## Discussion

In a retrospective study, we investigated the clinicopathological significance of TANs and CAFs in EGC. We revealed that TANs and CAFs, rather than peripheral blood neutrophil count and NLR, were positively associated with clinicopathological features including tumor size, Lauren classification, LVI, and LNM. In addition to histological classification and LVI, TANs were found to be an independent risk factor for LNM in all EGC patients. Stratification analysis by depth of invasion showed that CAFs in intramucosal tumor, LVI in SM1 tumor, both LVI and TANs in SM2 tumor were independent risk factors for LNM. Our results demonstrated that TANs can predict LNM in EGC.

Neutrophils are the most abundant inflammatory cells in human circulation, and neutrophils have been revealed to regulate tumor progression in recent years. TANs can be divided into N1 and N2 TANs according to their activation and cytokine status and effects on tumor cell growth. N1 TANs exert an antitumor activity, by direct or indirect cytotoxicity. N2 TANs stimulate immunosuppression, tumor growth, angiogenesis, and metastasis by DNA instability or by cytokine and chemokine release (22). Nuclear morphology was suggested to distinguish between these two subtypes, with N1 neutrophils having a hypersegmented nucleus and N2 neutrophils consisting of banded or ring-like nuclei (23), and there are no distinct markers to distinguish between the two states of TAN so far (24). In this study, TANs were found to be associated with LNM in EGC; however, we did not identify the subtype of TANs. High-level TANs in GC patients have been shown to be associated with disease progression and poor clinical outcome (12), but in Epstein–Barr virus-associated gastric carcinoma with abundant lymphocytic interstitium, a high density of CD66b-positive TANs is associated with intestinal-type histology and low frequency of LNM (25), and the presence of neutrophil infiltration in the corpus was closely related to the development of metachronous gastric cancer after ESD (26). Hence, how TANs promote LNM or tumor progression in gastric cancer remains unclear. We have observed neutrophils in the tumor thrombus in the tumor-draining lymphatics of gastric cancer (data not shown), which may be one of the underlying mechanisms and needs further investigation.

CAFs are the prominent cell component in the TME of gastric cancer, and CAFs can produce IL-8 to recruit neutrophils (14). A recent study indicated that the combination of CD66b+ TANs and α-SMA+ CAFs could be used as an independent factor for poor outcomes in gastric cancer patients (27). We also evaluated the clinicopathological significance of CAFs in addition to TANs in EGC. CAFs mainly existed in deep submucosal tumors (SM2), and its prevalence in the high-CAF subgroup was 90.5%. In SM2 tumors, it is difficult to identify the presence of LNM in endoscopic therapy for EGC (28). We showed that TANs, besides LVI, were an independent factor of LNM for SM2 tumors. LNM was found in 18.7% (14/75) in the low-TAN group, while it increased to 40.8% (20/49) in the high-TAN group. However, CAFs were not identified as an independent risk factor for LNM in our EGC cohort (*P* = 0.051). We further evaluated the maturation of CAFs in the TME. It has been shown that mature CAFs may produce more collagen I and IL-8 (29). Abundant collagen I increases the stiffness of tumor stroma and the internal pressure of the tumor, which results in the rupture of tumor cells and paves the way for the spread or metastasis of cancer cells together with the regular alignment of fibers (30, 31). The increased stromal stiffness aggravates hypoxic TME, which promotes neutrophil polarization and inhibits its apoptosis (32). Besides histological morphology, the CAF immunophenotypes are variable, and the level of α-SMA expression is higher in the immature CAF subtype than in the mature CAF subtype (33), as we have found. We further demonstrated that IL-8 was highly expressed in mature CAFs than in immature CAFs; that mature CAFs correlated with TANs in SM tumors, especially in SM2 tumors; and that mature CAFs were significantly associated with LNM in SM2 tumors. Therefore, we speculated that mature CAFs produce more IL-8, recruit more neutrophils to cancer tissues, and promote neutrophil polarization, which synergistically promotes LNM and tumor progression in EGC, especially in SM2 tumors.

The clinical significance of neutrophil counts and NLR in EGC remains uncertain (34, 35). We showed that both neutrophil counts and NLR had no significant association with the clinicopathological features except patient gender in our EGC cohort. The inconsistency among various reports may be due to different patient cohorts and different cutoffs of NLR, and further studies are needed to elucidate the clinicopathological and prognostic values.

Collectively, our results demonstrated that TANs predict LNM in EGC and that TANs may be incorporated into the current morphological criteria of endoscopic therapy for EGC, which will help to estimate LNM precisely. Of course, further prospective studies are needed to evaluate TANs as a marker of LNM in EGC. We have collected a few pathological data of EGC patients undergoing additional gastrectomy due to non-curative endoscopic treatment depending on the current criteria, and we intend to establish a comprehensive and quantitative predictive system for LNM.

## Data Availability Statement

The raw data supporting the conclusions of this article will be made available by the authors, without undue reservation.

## Ethics Statement

The studies involving human participants were reviewed and approved by Institutional Review Board of Nanjing University of Chinese Medicine (2019NL-098-02). The patients/participants provided their written informed consent to participate in this study.

## Author Contributions

LS, YW, and JZ conceived the study. YW, JZ, and TZ performed the experiments and drafted the manuscript. SH, YZ, and XY collected all tissue samples and clinical information. YZ supported the experimental techniques. LS reviewed the manuscript and provided financial support. All authors read and approved the final manuscript.

## Conflict of Interest

The authors declare that the research was conducted in the absence of any commercial or financial relationships that could be construed as a potential conflict of interest.
