# Supplementary figures and images for: Tumor-Associated Neutrophils Can Predict Lymph Node Metastasis in Early Gastric Cancer
Source: Front Oncol. 2020 Sep 21;10:570113. doi: 10.3389/fonc.2020.570113 (PMC7537418; doi:10.3389/fonc.2020.570113)

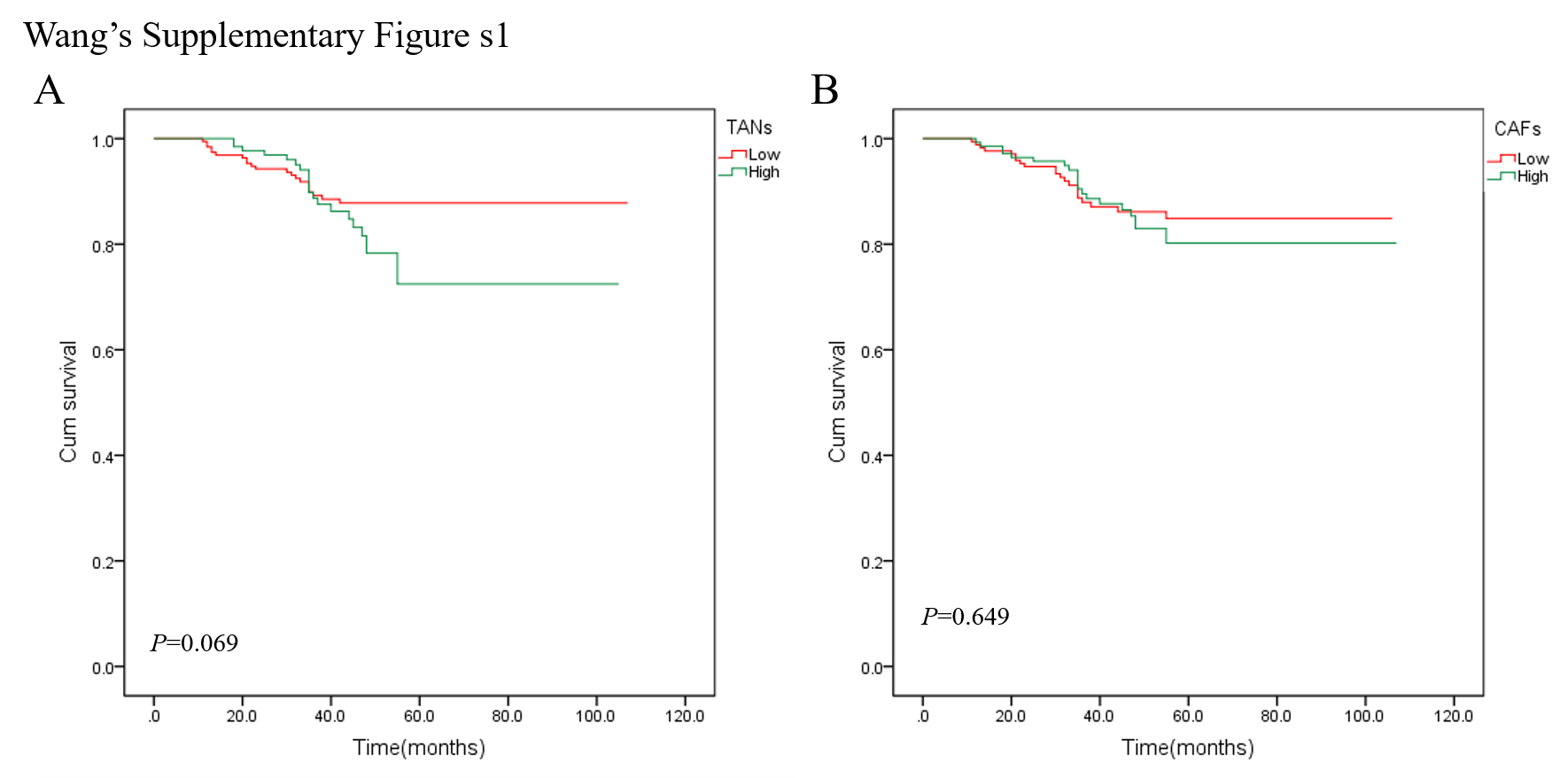

Supplement: Supplementary file 1 [file Image_1.TIF]
