# Supplementary material for: Tumor-Associated Neutrophils Can Predict Lymph Node Metastasis in Early Gastric Cancer
Source: Front Oncol. 2020 Sep 21;10:570113. doi: 10.3389/fonc.2020.570113 (PMC7537418; doi:10.3389/fonc.2020.570113)
Supplement: Supplementary file 2 [file Table_1.DOCX]

**Table s1. Correlation between TANs and CAFs in the early gastric cancer tissues.**

| **ALL** | | **CAFs** | | ***χ2*** | ***r*** | ***P*** |
| --- | --- | --- | --- | --- | --- | --- |
|  |  | **High** | **Low** |  |  |  |
| **TANs** | **High** | 60 | 63 | 0.850 | 0.059 | 0.351 |
|  | **Low** | 80 | 107 |  |  |  |

| **SM** | | **CAFs** | | ***χ2*** | ***r*** | ***P*** |
| --- | --- | --- | --- | --- | --- | --- |
|  |  | **High** | **Low** |  |  |  |
| **TANs** | **High** | 54 | 12 | 0.000 | -0.008 | 1.000 |
|  | **Low** | 75 | 16 |  |  |  |

| **SM1** | | **CAFs** | | ***χ2*** | ***r*** | ***P*** |
| --- | --- | --- | --- | --- | --- | --- |
|  |  | **High** | **Low** |  |  |  |
| **TANs** | **High** | 13 | 7 | 0.253 | 0.128 | 0.530 |
|  | **Low** | 11 | 10 |  |  |  |

| **SM2** | | **CAFs** | | ***χ2*** | ***r*** | ***P*** |
| --- | --- | --- | --- | --- | --- | --- |
|  |  | **High** | **Low** |  |  |  |
| **TANs** | **High** | 41 | 5 | 0.008 | -0.038 | 0.751 |
|  | **Low** | 64 | 6 |  |  |  |

*TANs* tumor-associated neutrophils, *CAFs* cancer-associated fibroblasts
