# Supplementary material for: Tumor-Associated Neutrophils Can Predict Lymph Node Metastasis in Early Gastric Cancer
Source: Front Oncol. 2020 Sep 21;10:570113. doi: 10.3389/fonc.2020.570113 (PMC7537418; doi:10.3389/fonc.2020.570113)
Supplement: Supplementary file 3 [file Table_2.DOCX]

**Table s2. The clinical significance of neutrophil count and neutrophil/lymphocyte ratio (****NLR) in the preoperative blood of the early gastric cancer patients (n=322).**

| **Clinicopathologic Features** | | **Neutrophil count (×10^9^/L)** | ***F*** | ***P*** | **NLR** | | ***χ^2^*** | ***P*** |
| --- | --- | --- | --- | --- | --- | --- | --- | --- |
|  |  |  |  |  | **≤1.9 (%)** | **＞1.9 (%)** |  |  |
| Gender | Male | 3.51±1.39 | 0.994 | 0.019 | 99 (45.2) | 120 (54.8) | 5.710 | 0.017 |
|  | Female | 3.13±1.32 |  |  | 62 (60.2) | 41 (39.8) |  |  |
| Age (year) | ＜65 | 3.42±1.37 | 0.011 | 0.612 | 105 (50.2) | 104 (49.8) | 0.000 | 1.000 |
|  | ≥ 65 | 3.34±1.38 |  |  | 56 (49.6) | 57 (50.4) |  |  |
| Tumor location in the stomach | Upper third | 3.29±1.10 | 1.022 | 0.361 | 39 (55.7) | 31 (44.3) | 2.200 | 0.333 |
|  | Middle third | 3.25±1.05 |  |  | 41 (53.2) | 36 (46.8) |  |  |
|  | Lower third | 3.49±1.58 |  |  | 81 (46.3) | 94 (53.7) |  |  |
| Tumor size (cm) | ≤ 2 | 3.40±1.15 | 0.548 | 0.578 | 86 (50.9) | 83 (49.1) | 0.127 | 0.955 |
|  | 2 - 2.9 | 3.47±1.52 |  |  | 44 (48.9) | 46 (51.1) |  |  |
|  | ≥ 3 | 3.24±1.23 |  |  | 31 (49.2) | 32 (50.8) |  |  |
| Macroscopic type | Elevated | 3.44±1.45 | 0.145 | 0.865 | 10 (38.5) | 16 (61.5) | 1.598 | 0.442 |
|  | Flat | 3.29±0.98 |  |  | 25 (53.2) | 22 (46.8) |  |  |
|  | Depressed | 3.40±1.43 |  |  | 126 (50.6) | 123 (49.4) |  |  |
| Depth of invasion | Intramucosal | 3.36±1.33 | 0.071 | 0.932 | 77 (49.4) | 79 (50.6) | 1.828 | 0.410 |
|  | SM1 | 3.36±1.25 |  |  | 25 (59.5) | 17 (40.5) |  |  |
|  | SM2 | 3.42±1.47 |  |  | 59 (47.6) | 65 (52.4) |  |  |
| Lauren classification | Intestinal | 3.39±1.37 | 1.044 | 0.373 | 94 (50.5) | 92 (49.5) | 1.511 | 0.685 |
|  | Diffuse | 3.25±1.21 |  |  | 24 (49.0) | 25 (51.0) |  |  |
|  | Mixed | 3.57±1.55 |  |  | 34 (46.6) | 39 (53.4) |  |  |
|  | Not defined | 2.95±0.69 |  |  | 9 (64.3) | 5 (35.7) |  |  |
| Histolological classification | Well | 3.38±1.76 | 0.281 | 0.755 | 25 (58.1) | 18 (41.9) | 1.442 | 0.484 |
|  | Moderately | 3.44±1.29 |  |  | 76 (47.8) | 83 (52.2) |  |  |
|  | Poorly | 3.32±1.33 |  |  | 60 (50.0) | 60 (50.0) |  |  |
| Lymphovascular invasion | Absence | 3.40±1.35 | 0.431 | 0.759 | 130 (48.3) | 139 (51.7) | 1.445 | 0.229 |
|  | Presence | 3.34±1.48 |  |  | 31 (58.5) | 22 (41.5) |  |  |
| Perineural invasion | Absence | 3.40±1.38 | 0.892 | 0.544 | 154 (50.0) | 154 (50.0) | 0.000 | 1.000 |
|  | Presence | 3.17±1.14 |  |  | 7 (50.0) | 7 (50.0) |  |  |
| *H. pylori* infection | Absence | 3.36±1.34 | 0.128 | 0.581 | 115 (51.3) | 109 (48.7) | 0.367 | 0.545 |
|  | Presence | 3.45±1.45 |  |  | 46 (46.9) | 52 (53.1) |  |  |
| Lymph node metastasis | Absence | 3.42±1.36 | 0.129 | 0.393 | 136 (49.8) | 137 (50.2) | 0.000 | 1.000 |
|  | Presence | 3.23±1.43 |  |  | 25 (51.0) | 24 (49.0) |  |  |

*NLR* neutrophil-to-lymphocyte ratio
