# Supplementary material for: Tumor-Associated Neutrophils Can Predict Lymph Node Metastasis in Early Gastric Cancer
Source: Front Oncol. 2020 Sep 21;10:570113. doi: 10.3389/fonc.2020.570113 (PMC7537418; doi:10.3389/fonc.2020.570113)
Supplement: Supplementary file 4 [file Table_3.DOCX]

**Table s3. Clinicopathologic features associated with lymph node metastasis (LNM) in the patients with intramucosal early gastric cancer.**

| **Clinicopathologic Features** | | **LNM** | | ***χ^2^*** | ***P*** |
| --- | --- | --- | --- | --- | --- |
|  |  | **Present**  **(n=9) (%)** | **Absent**  **(n=147) (%)** |  |  |
| Gender | Male | 7 (6.9) | 94 (93.1) | 0.234 | 0.494 |
|  | Female | 2 (3.6) | 53 (96.4) |  |  |
| Tumor location in the stomach | Upper third | 0 (0.0) | 29 (100.0) | 2.178 | 0.354 |
|  | Middle third | 3 (8.3) | 33 (91.7) |  |  |
|  | Lower third | 6 (6.6) | 85 (93.4) |  |  |
| Age (year) | ＜65 | 5 (4.5) | 107 (95.5) | 0.538 | 0.271 |
|  | ≥ 65 | 4 (9.1) | 40 (90.9) |  |  |
| Tumor size (cm) | ＜2 | 3 (2.9) | 101 (97.1) | 5.572 | 0.038 |
|  | 2 - 2.9 | 5 (13.9) | 31 (86.1) |  |  |
|  | ≥ 3 | 1 (6.3) | 15 (93.8) |  |  |
| Macroscopic type | Elevated | 0 (0.0) | 7 (100.0) | 2.102 | 0.350 |
|  | Flat | 1 (3.1) | 31 (96.9) |  |  |
|  | Depressed | 8 (6.8) | 109 (93.2) |  |  |
| Lauren classification | Intestinal | 2(2.1) | 94 (97.9) | 9.711 | 0.004 |
|  | Diffuse | 1 (3.6) | 27 (96.4) |  |  |
|  | Mixed | 6 (18.8) | 26 (81.3) |  |  |
|  | Not defined | 0 (21.4) | 0 (78.6) |  |  |
| Histolological classification | Well | 0 (0.0) | 19 (100.0) | 7.868 | 0.015 |
|  | Moderately | 1 (1.4) | 73 (98.6) |  |  |
|  | Poorly | 8 (12.7) | 55 (87.3) |  |  |
| Lymphovascular invasion | Absence | 9(5.9) | 144 (94.1) | 0.000 | 1.000 |
|  | Presence | 0 (0.0) | 3 (100.0) |  |  |
| Perineural invasion | Absence | 9 (5.8) | 147 (94.2) | 0.000 | 1.000 |
|  | Presence | 0 (0.0) | 0 (0.0) |  |  |
| *H. pylori* infection | Absence | 8 (7.1) | 104 (92.9) | 0.628 | 0.447 |
|  | Presence | 1 (2.3) | 43 (97.7) |  |  |
| TANs | Low | 2 (2.1) | 94 (97.9) | 4.599 | 0.028 |
|  | High | 7 (11.7) | 53 (88.3) |  |  |
| CAFs | High | 4 (36.4) | 7 (63.6) | 12.130 | 0.002 |
|  | Low | 5 (3.5) | 137(96.5) |  |  |
| Neutrophil count | average±SD | 3.34±1.50 | 3.37±1.33 | F=0.491 | 0.947 |
| NLR | Low (≤1.9) | 3 (3.9) | 74 (96.1) | 0.419 | 0.495 |
|  | High (＞1.9) | 6 (7.6) | 73 (92.4) |  |  |

*LNM* lymph node metastasis, *TANs* tumor-associated neutrophils, *CAFs* cancer-associated fibroblasts, *NLR* neutrophil-to-lymphocyte ratio
