# Supplementary material for: Tumor-Associated Neutrophils Can Predict Lymph Node Metastasis in Early Gastric Cancer
Source: Front Oncol. 2020 Sep 21;10:570113. doi: 10.3389/fonc.2020.570113 (PMC7537418; doi:10.3389/fonc.2020.570113)
Supplement: Supplementary file 5 [file Table_4.DOCX]

**Table s4. Multivariate logistic regression analysis of potential risk factors for lymph node metastasis in the patients with intramucosal early gastric cancer.**

| **Clinicopathologic Features** | **Odds ratio** | **95% confidence interval** | ***P*** |
| --- | --- | --- | --- |
| CAFs | 12.018 | 1.652-87.446 | 0.049 |

*CAFs* cancer-associated fibroblasts
