# Supplementary material for: Tumor-Associated Neutrophils Can Predict Lymph Node Metastasis in Early Gastric Cancer
Source: Front Oncol. 2020 Sep 21;10:570113. doi: 10.3389/fonc.2020.570113 (PMC7537418; doi:10.3389/fonc.2020.570113)
Supplement: Supplementary file 6 [file Table_5.DOCX]

**Table s5. Clinicopathologic features associated with lymphnode metastasis (LNM) in the patients with submucosal early gastric cancer.**

| **Clinicopathologic Features** | | **LNM** | | ***χ^2^*** | ***P*** |
| --- | --- | --- | --- | --- | --- |
|  |  | **Present**  **(n=40) (%)** | **Absent**  **(n=126) (%)** |  |  |
| Gender | Male | 23 (19.5) | 95 (80.5) | 3.901 | 0.044 |
|  | Female | 17 (35.4) | 31 (64.6) |  |  |
| Tumor location in the stomach | Upper third | 7 (17.1) | 34 (82.9) | 2.020 | 0.367 |
|  | Middle third | 9 (22.0) | 32 (78.0) |  |  |
|  | Lower third | 24 (28.6) | 60 (71.4) |  |  |
| Age (year) | ＜65 | 21 (21.6) | 76 (78.4) | 0.476 | 0.462 |
|  | ≥ 65 | 19 (27.5) | 50 (72.5) |  |  |
| Tumor size (cm) | ＜2 | 12 (18.5) | 53 (81.5) | 2.684 | 0.255 |
|  | 2 - 2.9 | 13 (24.1) | 41 (75.9) |  |  |
|  | ≥ 3 | 15 (31.9) | 32 (68.1) |  |  |
| Macroscopic type | Elevated | 5 (26.3) | 14 (73.7) | 0.220 | 0.948 |
|  | Flat | 3 (20.0) | 12 (80.0) |  |  |
|  | Depressed | 32 (24.2) | 100 (75.8) |  |  |
| Lauren classification | Intestinal | 12 (13.3) | 78 (86.7) | 16.337 | 0.001 |
|  | Diffuse | 6 (28.6) | 15 (71.4) |  |  |
|  | Mixed | 19 (46.3) | 22 (53.7) |  |  |
|  | Not defined | 3 (21.4) | 11 (78.6) |  |  |
| Histolological classification | Well | 2 (8.3) | 22 (91.7) | 10.615 | 0.004 |
|  | Moderately | 16 (18.8) | 69 (81.2) |  |  |
|  | Poorly | 22 (38.6) | 35 (61.4) |  |  |
| Lymphovascular invasion | Absence | 9 (7.8) | 107 (92.2) | 53.277 | 0.000 |
|  | Presence | 31 (62.0) | 19 (38.0) |  |  |
| Perineural invasion | Absence | 34 (22.4) | 118 (77.6) | 1.929 | 0.104 |
|  | Presence | 6 (42.9) | 8 (57.1) |  |  |
| *H. pylori* infection | Absence | 25 (22.3) | 87 (77.7) | 0.332 | 0.562 |
|  | Presence | 15 (27.8) | 39 (72.2) |  |  |
| TANs | Low | 15 (15.6) | 81 (84.4) | 7.868 | 0.003 |
|  | High | 25 (35.7) | 45 (64.3) |  |  |
| CAFs | High | 35 (27.1) | 94 (72.9) | 10.443 | 0.003 |
|  | Low | 1 (3.6) | 27 (96.4) |  |  |
| Neutrophil count | average±SD | 3.21±1.43 | 3.47±1.41 | F=1.108 | 0.312 |
| NLR | Low (≤1.9) | 22 (26.2) | 62 (73.8) | 0.209 | 0.588 |
|  | High (＞1.9) | 18 (22.0) | 64 (78.0) |  |  |

*LNM* lymph node metastasis, *TANs* tumor-associated neutrophils, *CAFs* cancer-associated fibroblasts, *NLR* neutrophil-to-lymphocyte ratio
