# Supplementary material for: Tumor-Associated Neutrophils Can Predict Lymph Node Metastasis in Early Gastric Cancer
Source: Front Oncol. 2020 Sep 21;10:570113. doi: 10.3389/fonc.2020.570113 (PMC7537418; doi:10.3389/fonc.2020.570113)
Supplement: Supplementary file 7 [file Table_6.DOCX]

**Table s6. Multivariate logistic regression analysis of potential risk factors for lymph node metastasis in the patients with SM1 early gastric cancer.**

| **Clinicopathologic Features** | **Odds ratio** | **95% confidence interval** | ***P*** |
| --- | --- | --- | --- |
| Lymphovascular invasion | 16.086 | 1.114-232.184 | 0.041 |
