# Supplementary material for: Tumor-Associated Neutrophils Can Predict Lymph Node Metastasis in Early Gastric Cancer
Source: Front Oncol. 2020 Sep 21;10:570113. doi: 10.3389/fonc.2020.570113 (PMC7537418; doi:10.3389/fonc.2020.570113)
Supplement: Supplementary file 8 [file Table_7.DOCX]

**Table s7. Correlation between TANs and mature or immature CAFs in SM1 gastric cancer tissues (n=24).**

| **SM1** | | **CAFs** | | ***χ2*** | ***r*** | ***P*** |
| --- | --- | --- | --- | --- | --- | --- |
|  |  | **mature** | **immature** |  |  |  |
| **TANs** | **High** | 1 | 12 | 0.000 | 0.192 | 1.000 |
|  | **Low** | 0 | 11 |  |  |  |

*TANs* tumor-associated neutrophils, *CAFs* cancer-associated fibroblasts
