# Supplementary material for: Tumor-Associated Neutrophils Can Predict Lymph Node Metastasis in Early Gastric Cancer
Source: Front Oncol. 2020 Sep 21;10:570113. doi: 10.3389/fonc.2020.570113 (PMC7537418; doi:10.3389/fonc.2020.570113)
Supplement: Supplementary file 9 [file Table_8.DOCX]

**Table s8. The clinical relevance of the maturity of CAFs in SM2 gastric cancers.**

| **Clinicopathologic Features** | | **CAFs** | | **χ^2^** | ***P*** |
| --- | --- | --- | --- | --- | --- |
|  |  | **immature**  **(n=81) (%)** | **mature**  **(n=24) (%)** |  |  |
| Gender | Male | 59 (80.8) | 14 (19.2) | 1.218 | 0.210 |
|  | Female | 22 (68.8) | 10 (31.3) |  |  |
| Age (year) | ＜65 | 48 (81.4) | 11 (18.6) | 0.865 | 0.254 |
|  | ≥ 65 | 33 (71.7) | 13 (28.3) |  |  |
| Tumor location in the stomach | Upper third | 13 (61.9) | 8 (38.1) | 3.269 | 0.174 |
|  | Middle third | 21 (80.8) | 5 (19.2) |  |  |
|  | Lower third | 47 (81.0) | 11 (19.0) |  |  |
| Tumor size (cm) | ＜2cm | 32 (76.2) | 10 (23.8) | 0.234 | 0.916 |
|  | 2 - 2.9 | 25 (75.8) | 8 (24.2) |  |  |
|  | ≥ 3 | 24 (80.0) | 6 (20.0) |  |  |
| Macroscopic type | Elevated | 6 (66.7) | 3 (33.3) | 3.009 | 0.262 |
|  | Flat | 6 (60.0) | 4 (40.0) |  |  |
|  | Depressed | 69 (80.2) | 17 (19.8) |  |  |
| Lauren’s classification | Intestinal | 48 (84.2) | 9 (15.8) | 4.881 | 0.166 |
|  | Diffuse | 11 (73.3) | 4 (26.7) |  |  |
|  | Mixed | 17 (70.8) | 7 (29.2) |  |  |
|  | Not defined | 5 (55.6) | 4 (44.4) |  |  |
| Histolological classification | Well | 12 (92.3) | 1 (7.7) | 4.626 | 0.105 |
|  | Moderately | 44 (81.5) | 10 (18.5) |  |  |
|  | Poorly | 25 (65.8) | 13 (34.2) |  |  |
| Lymphovascular invasion | Absence | 55 (83.3) | 11 (16.7) | 2.975 | 0.058 |
|  | Presence | 26 (66.7) | 13 (33.3) |  |  |
| Perineural invasion | Absence | 71 (76.3) | 22 (23.7) | 0.031 | 0.729 |
|  | Presence | 10 (83.3) | 2 (16.7) |  |  |
| *H. pylori* infection | Absence | 55 (76.4) | 17 (23.6) | 0.000 | 1.000 |
|  | Presence | 26 (78.8) | 7 (21.2) |  |  |
| Lymphnode metastasis | Absence | 64 (85.3) | 11 (14.7) | 8.427 | 0.004 |
|  | Presence | 17 (56.7) | 13 (43.3) |  |  |
| Neutrophil count | average±SD | 3.50±1.51 | 3.24±1.24 | F=0.346 | 0.438 |
| NLR | Low (≤1.9) | 34 (72.3) | 13 (27.7) | 0.674 | 0.353 |
|  | High (＞1.9) | 47 (81.0) | 11 (19.0) |  |  |

*CAFs* cancer-associated fibroblasts, *NLR* neutrophil-to-lymphocyte ratio
